# Psychometric Properties and Cross-Cultural Invariance of the Beck Depression Inventory-II and Beck Anxiety Inventory among a Representative Sample of Spanish, Portuguese, and Brazilian Undergraduate Students

**DOI:** 10.3390/ijerph20116009

**Published:** 2023-05-31

**Authors:** Rodrigo Leão Ferreira do Nascimento, Fernando Fajardo-Bullon, Eduardo Santos, J. Landeira-Fernandez, Luis Anunciação

**Affiliations:** 1 Department of Psychology, Pontifical Catholic University of Rio de Janeiro (PUC-Rio), Rio de Janeiro 22453-900, Brazil; 2 Department of Psychology, Faculty of Education and Psychology, University of Extremadura, 06006 Badajoz, Spain; 3 Department of Psychology, Faculty of Psychology and Educational Sciences, University of Coimbra, 3004-531 Coimbra, Portugal

**Keywords:** measurement invariance, depression, anxiety, Multigroup Confirmatory Factor Analysis

## Abstract

Clinical psychologists often use the Beck Depression Inventory, 2nd edition (BDI-II), and Beck Anxiety Inventory (BAI) to aid in the diagnosis of mental health issues and verify the effectiveness of treatments. Despite this common practice, studies that implement a cross-cultural design to check psychometric properties and the invariance of these scales are still scarce in the literature, which can lead to biased results that prevent comparisons among different groups. The present study investigated the internal structure of both tools and their level of invariance. From a representative sample of undergraduate students from Spain (*n* = 1216), Portugal (*n* = 426), and Brazil (*n* = 315), Confirmatory Factor Analysis and Multigroup Confirmatory Factor Analysis were performed. The results revealed suitable fit indices for the two-factor structure of the BDI-II and BAI, assessed by Confirmatory Factor Analysis procedures. Additionally, the two-factor model of the BDI-II reached invariant properties at three levels, whereas the structural model of the BAI did not. Altogether, these results suggest using the BDI-II in this group in these three countries and imply that BAI scores should be interpreted cautiously.

## 1. Introduction

Depression and anxiety are clinically important disorders with a high prevalence among psychiatric conditions worldwide [1]. With the emergence of the COVID-19 pandemic, the prevalence increased to 246 million (3153 cases per 100,000) for major depressive disorder and 374 million (4802 per 100,000) for anxiety disorders [2]. According to the World Mental Health Report (World Health Organization, 2022) [3], high- and low-income countries are affected by mental health conditions, especially women and younger groups. Different actions must be taken to address this situation, including clinical assessments that are supported by tools with adequate psychometric properties.

Among the different instruments that are used to aid the diagnosis of depression and anxiety, the Beck Depression Inventory, 2nd edition (BDI-II), and Beck Anxiety Inventory (BAI) are widely cited in the literature [4,5,6]. In 1961, Dr. Aaron T. Beck and his colleagues developed the initial version of the BDI. This instrument underwent a reformulation in 1978. Subsequently, in 1996, the second edition of the BDI (BDI-II) was introduced, which incorporated symptoms that were outlined in the Diagnostic and Statistical Manual of Mental Disorders, 4th edition (DSM-IV) [4]. The current study employed factor analysis to examine the underlying structure of the BDI-II in two separate samples: outpatients and college students. The results revealed the presence of two factors in both samples. The first group of participants yielded factors that referred to “Somatic-Affective” and “Cognitive”, and the second group yielded factors that referred to “Cognitive-Affective” and “Somatic.” The authors of the study proposed that affective items, such as “Sadness” and “Crying”, that are present in the BDI-II may vary relative to the characteristics of the sample. Overall, however, the factorial solution of two positively correlated dimensions (Cognitive-Affective and Somatic) was proposed as the most fitting representation of the data [7].

Widespread global acceptance of the BDI-II has led to a growing interest in investigating its factorial structure, which has been found to vary depending on the type of sample and the number of factors that are considered [8,9]. A recent study that was conducted with a community population revisited some prominent factorial models of the BDI-II, examining solutions that ranged from two-dimensional to bi-factorial, with a further consideration of three-dimensional models. The diversity of factorial solutions that are obtained in the current study highlights the challenge of determining an optimal and generalizable model for the BDI-II [8]. Additionally, the complexity of the issue is further compounded by the fact that depressive symptoms appear to differ across cultures. Studies have been inconclusive with regard to whether depression is perceived as more psychological (cognitive) in nature among Western individuals compared with Eastern individuals, who tend to view it as more somatic in nature. This observation highlights the need to consider cultural variations when examining the factorial structure of the BDI-II and other measures of depression [9,10].

Anxiety symptoms are evaluated by different instruments, mainly self-report items. Nevertheless, only the seminal article by Beck, Epstein, Brown, and Steer (1988) [11] achieved discriminant validity that was able to differentiate anxiety from depressive symptoms. In their study, the BAI exhibited high reliability (α = 0.92). In 1993, Beck and Steer conducted a revision and re-examination of the evidence, proposing a two-dimensional factorial structure, referred to as “Somatic Symptoms” and “Subjective Affective and Panic Symptoms”, for the instrument in question. Since then, the instrument has gained significant popularity, leading to numerous studies that investigated its factorial structure [12,13,14,15]. A review by Bardoshi and colleagues (2016) on the psychometric properties of the instrument found evidence that supports a two-factor structure, although the fit indices did not necessarily meet the recommended cut-off points in the literature. Additionally, this study revealed that the instrument had high levels of internal consistency for nonclinical samples (α = 0.91), indicating its good reliability [15]. Nonetheless, there is no unanimity in the literature about the factorial composition of the BAI. Some studies suggest a general factor, whereas others propose a multidimensional model [14,15,16,17]. Additionally, the suitability of the lexicon that is employed for BAI items has been contested. A recent study that examined Latinos who live in the United States, for example, raised concerns about the various terms that are employed in the BAI that may be interpreted distinctively among the diverse ethnic groups that are represented within that population [18]. Consequently, although the BAI and BDI have robust psychometric properties and are extensively utilized, factorial and cultural concerns continue with regard to their application.

The extensive use of these instruments with clinical and nonclinical populations in different countries has further revealed their importance but also led to concerns about measurement invariance. According to Dere et al. (2015) [9], many health professionals assume they are assessing the same construct across populations in the same way. They assume that the measure that is assessed is invariant across groups. However, this assumption must be verified. There is a scarcity of studies on the psychometric properties of these instruments in developing countries (e.g., Brazil) in general [19]. Fostering further studies in this area is important. Only two studies that assessed the invariance of the BDI-II were found for Brazilian samples [8,20]. We found no study that assessed the invariance of the BAI for a similar group. Given the significance of the topic and ongoing debate about the factorial structure of the instruments in question, the present study investigated the initial models that were proposed by Beck using Confirmatory Factor Analysis (CFA) techniques (multigroup). The assessment of clinical models with nonclinical populations has been investigated in the literature [21]. Furthermore, gathering evidence about the factorial structure of the instrument may be relevant to explore a vast clinical symptomatology with a vulnerable group. Thus, to explore the generalizability of the two-factor solution that was identified by the author across various cultures, we adopted the factorial model that was identified for the clinical patient sample while utilizing the BDI-II. By conducting a thorough examination of these models, the present study provides further evidence of the psychometric properties of these instruments, thereby advancing the ongoing debate on the topic. The present study investigated the invariance of the BDI-II and BAI in a representative sample of undergraduate students from Spain, Portugal, and Brazil.

## 2. Materials and Methods

### 2.1. Participants and Procedures

The data were collected in three different settings (University of Extremadura, Spain; University of Coimbra, Portugal; Pontifical Catholic University of Rio de Janeiro [PUC-Rio], Brazil) in 2015. Stratified probability sampling was performed, which makes the total participants representative of the three institutions. More details about the sampling procedures can be found elsewhere [22].

The final analytic sample consisted of 1957 undergraduate students. Of these, 62.1% (*n* = 1210) were from the University of Extremadura (Spain), 21.8% (*n* = 426) were from the University of Coimbra (Portugal), and 16.1% (*n* = 293) were from PUC-Rio (Brazil). The mean ages were 21.49 years (SD = 3.02 years) for the Spanish students, 20.43 years (SD = 1.66 years) for the Portuguese students, and 22.83 years (SD = 7.18 years) for the Brazilian students.

A team of trainees and previously trained professionals performed the data collection. To control for eventual bias, the questionnaires were completed between school exam periods. All participants were informed about the study objectives, and their further questions were answered by the research team. Ethical approval was obtained from the local Research Ethics Committees in the respective countries.

### 2.2. Measures

#### 2.2.1. Beck Depression Inventory-II

The BDI (Beck et al., 1961) [23] has been extensively used to assess and screen for depressive symptoms in nonclinical and clinical populations [15]. With the release of the DSM-III-R and DSM-IV, the authors revised the BDI, producing the 2nd edition (BDI-II; Beck et al., 1996) [24]. Since then, many reviews have shown its good psychometric properties [8,22,23,24,25,26]. For the BDI-II, participants rate different symptoms on a Likert-type scale, ranging from 0 to 3. A total score is calculated by summing the ratings for all 21 items. This instrument has presented evidence of validity from studies in Spain [17], Portugal [27], and Brazil [28]. These studies showcased optimal results, such as the α for the BDI-II equals 0.89 (95% confidence interval [CI] = 0.89–0.90).

#### 2.2.2. Beck Anxiety Inventory

The BAI (Beck et al., 1988) [11] is a self-report measure that is recommended for different populations and has good psychometric properties [29]. It consists of 21 items that assess the severity of anxiety symptoms on a Likert-type scale, ranging from 0 to 3. Studies have shown that the instrument has evidence of validity in Brazil [28], Portugal [16], and Spain [17]. Similar to a previous study [22], the α for the BAI was 0.90 (95% CI: 0.90–0.91).

### 2.3. Statistical Plan

The data analysis was conducted using a two-fold method. First, CFA was performed by assessing different BDI-II and BAI models [24,30]. A *χ*^2^ test [31] was used to explore fit index differences between the tested models. We also conducted a Multi-Group Factorial Analysis (MGCFA) to check whether invariance was achieved within the selected models in the different countries.

We performed two factorial models for each instrument, checking unidimensional and two-factor structures for the instruments. Because of the ordinal nature of the data, the psychometric analyses were based on the Robust Diagonally Weighted Least Squares as the estimator [32].

The threshold values to assess goodness of fit were the following: Comparative Fit Index (CFI) and Tucker–Lewis index (TLI) greater than 0.90 or 0.95 and Root Mean Square Error of Approximation (RMSEA) less than 0.08 or 0.06, with the upper limit of the CI less than 0.10 [33]. In accordance with previous literature [33], a *χ*^2^ test was conducted to check significant differences in fit indices between the factorial solutions.

The reliability of the selected models was estimated by the ordinal Cronbach alpha (α) and McDonald omega (ϖ) [34].

In the second part of the data analysis, an MGCFA was used to test the invariance of the BDI-II and BAI for Brazilian, Spanish, and Portuguese undergraduate students. We used the same aforementioned estimation method and fit indices, with the inclusion of the ΔCFI (less than 0.01) as recommended by Cheung and Rensvold (2002) [35]. The MGCFA assessed three levels of invariance: configural (factor structure), metric (loadings), and scalar (intercept). R Studio 4.02 software was used for all analyses, with the lavaan package (version 0.6.14).

## 3. Results

### 3.1. Preliminary Analyses

#### 3.1.1. Descriptive Analyses

Due to their skewness (SI) and kurtosis (KI) indicators surpassing the corresponding thresholds, typically greater than 2 and 7, respectively, for many items [36], BDI-II and BAI scores were non-normally distributed (Table 1). The missing-value inspection did not reveal any inconsistencies (approximately < 1.2%) for all items of both instruments.

#### 3.1.2. Sample Characteristics

A sample of 1957 participants was assessed in Spain (62.1%), Portugal (21.8%), and Brazil (16.1%). The mean age of the participants was 21.5 years (SD = 3.8 years), but significant differences were found between the three countries (*F*_2,1926_ = 35.19, *p* < 0.001). Significant differences were found in the proportions of males and females between Portuguese and Brazilian students compared with Spanish students (*χ*^2^_1_ = 161, *p* < 0.001). The values were similar to the results of [22] (See Table 2).

### 3.2. Evidence of Internal Structure

#### 3.2.1. BDI-II Results

**Figure 1:**
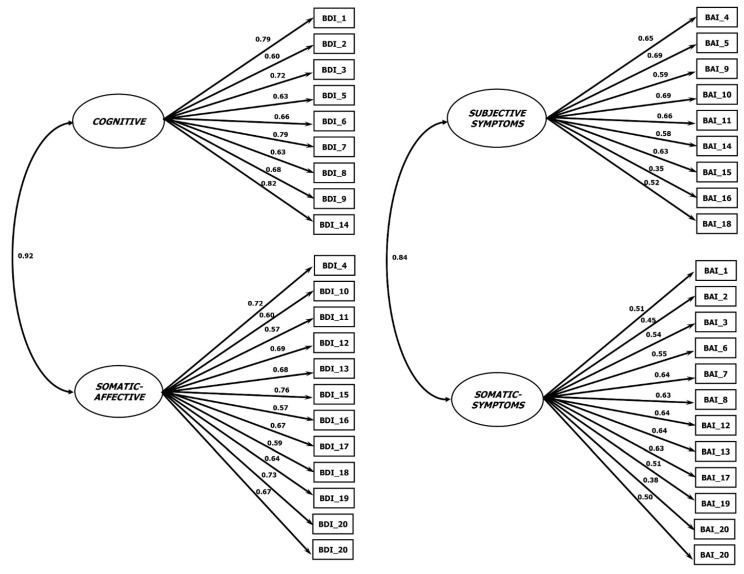
Factorial solutions of the BDI-II and BAI. The right model refers to the BDI-II and the left model refers to the BAI. “Subjective Symptoms” refers to “Subjective Affective and Panic Symptoms”.

The unifactorial model did not show satisfactory fit indices. The *χ*^2^ test value was significant (*χ*^2^_189_ = 913.126, *p* < 0.001). The RMSEA values were adequate (0.045; 90% CI = 0.042–0.048). The CFI (0.787) and TLI (0.764) values were not adequate. Different results were achieved with the two-factor model (Figure 1). The factor loadings values are shown in Table 3. The *χ*^2^ test value was significant (*χ*^2^_210_ = 3613.885, *p* < 0.001). The RMSEA values were adequate (0.037; 90% CI = 0.034–0.040). The CFI (0.855) and TLI (0.838) values were slightly below the recommended values (Table 4).

The goodness-of-fit indices are presented in Table 4. The results of the *χ*^2^ test [31] revealed that the two-factor model better fit the data compared with the unifactorial solution (*χ*^2^_1_ = 125.54, *p* < 0.001), which was previously expected. The reliability of the two-factor model revealed adequate values for both factors of the instrument: “Cognitive” (α = 0.81, ϖ = 0.82) and “Somatic-Affective” (α = 0.84, ϖ = 0.84).

#### 3.2.2. BAI Results

The BAI results partially followed the same pattern as in the previous analyses. The *χ*^2^ test value was significant (*χ*^2^_189_ = 1026.066, *p* < 0.001). The RMSEA values were adequate (0.049; 90% CI = 0.046–0.051). The CFI (0.777) and TLI (0.753) values were outside the current range adopted for cut-offs. Similar results were found for the two-factor model (Figure 1). The *χ*^2^ test value was significant (*χ*^2^_188_ = 971.658, *p* < 0.001). The RMSEA values were adequate (0.47; 90% CI = 0.044–0.050). The CFI and TLI values were not close to the prescribed values (0.792 and 0.767, respectively). The factor loadings are shown in Table 5.

The goodness-of-fit indices are presented in Table 6. The *χ*^2^ test [31] showed better results for the two-factor model compared with the unifactorial structure (*χ*^2^_1_ = 26.854, *p* < 0.001). The reliability analysis of the two-factor model revealed adequate results for both factors. For the “Somatic Symptoms” factor, the values were α = 0.84 and ϖ = 0.84. For the “Subjective Affective and Panic Symptoms” factor, the values were α = 0.82 and ϖ = 0.83.

### 3.3. Measurement Invariance

We performed an MGCFA to evaluate the invariance of the two-factor models of the BDI-II and BAI for Brazilian, Spanish, and Portuguese undergraduate students. The goodness-of-fit indices are presented in Table 7. The BDI-II model exhibited good indices at different invariance levels. The *χ*^2^ test was significant at the configural, metric, and scalar levels (*p* < 0.001). The RMSEA was 0.022 (CI = 0.018–0.026). The CFI and TLI values were 0.942 and 0.943, respectively. The ΔCFI (0.012) was slightly above the adopted cut-off point (0.01) [26]. Contrary to the previous results, the two-factor model of the BAI did not show invariance at any level. The *χ*^2^ test was significant at the configural, metric, and scalar levels (*p* < 0.001). The RMSEA was 0.035 (CI = 0.031–0.038). The CFI and TLI values were 0.863 and 0.865, respectively. The ΔCFI value was −0.039 at the scalar level.

## 4. Discussion

The main goal of the present study was to investigate the invariance of the BAI and BDI-II. This study was performed with a representative sample of undergraduate students from Brazil, Portugal, and Spain. All of the estimated BDI-II models showed good psychometric properties, such as good reliability values and good standard fit indices. The examined factorial models of the BAI did not demonstrate the anticipated outcomes. After comparing different solutions for each instrument, the two-factor models were sorted. These models exhibited high reliability levels, described by many coefficient estimators. Additionally, the BDI-II showed invariant results at three different levels: configural, metric, and scalar. In contrast to the aforementioned findings, the results of the BAI analysis were not consistent across the three levels of measurement (i.e., configural, metric, and scalar) and thus did not demonstrate invariance properties among the respective samples.

Several confirmatory models (unidimensional and two-factor by country and with the pooled sample) were performed to assess the BDI-II. The results of the factorial models revealed favorable fit indices. In cases in which multiple models are found to be suitable, a challenge arises in terms of determining the most appropriate factorial solution. A thorough examination of the factor loadings across countries and in the total sample revealed that all items exhibited loadings greater than 0.40 on their respective factors, with the exception of the “Loss of sexual interest” and “Feeling of punishment” items for the Brazilian sample (0.33 and 0.36, respectively) and “Suicidal thoughts” item for the Spanish (0.36) and Portuguese (0.34) samples. Altogether, these accumulated results indicated a robust factor structure. An analysis of the loadings for specific items revealed some discrepancies in factor loadings across different countries. Specifically, the “Loss of sexual interest” item in the “Somatic-Affective” factor displayed notable variability in loading, with a correlation of 0.33 for the Brazilian sample and correlations that ranged from 0.46 to 0.50 for the Spanish and Portuguese samples. Concerns about the lack of salience in this item were raised in the meta-analysis that was conducted by Huang et al. (2015) for the total sample and some study subgroups. Furthermore, the “Indecision” item exhibited divergent characteristics across countries. In Brazil, the factor loading was 0.50, whereas the respective factor loadings were 0.61 and 0.62 in Spain and Portugal. Additionally, the “Irritability” item displayed a lower correlation in the Brazilian sample (0.49) compared with the Spanish and Portuguese samples (0.60). Furthermore, the “Feeling of punishment” item in the “Cognitive” factor obtained a correlation of 0.36 in the Brazilian sample, 0.50 in the Spanish sample, and 0.47 in the Portuguese sample. Similarly, the “Suicidal thoughts” item displayed disparate behavior among the three countries. In Brazil, the factor loading was 0.55, whereas the corresponding factor loadings were 0.36 and 0.34 in Spain and Portugal, respectively. Altogether, this highlights the need for further examination and analysis to make a definitive selection [37].

Although there were discrepancies in factor loadings of specific items in the BDI-II, their values for the overall sample were prominent, with all items exhibiting a factor loading greater than 0.40. Moreover, the discussion that was proposed by a recent meta-analysis [25] about inconsistent findings for the factorial model structure of the BDI-II was extensively considered. In this previous study, the results supported the two-correlated-factor model that was proposed by Beck et al. (1996) [38]. Thus, a *χ*^2^ test [32] was performed to investigate differences between our models (*p* < 0.001). Considering such results, the two-factor solution was selected in our study. Reliability analyses were performed for the different factors (“Somatic-Affective” and “Cognitive”), supporting the internal consistency of the instrument.

A thorough examination of factor loadings across countries and in the total sample also revealed that all items of the BAI exhibited loadings greater than 0.40 on their respective factors, with the exception of the “Numbness or tingling” item for the Brazilian sample (0.32), “Face flushed” item for the Spanish sample (0.38), and “Fear of dying” item for the Spanish (0.32), Portuguese (0.32), and overall (0.35) samples. The analysis of loadings for specific items in the “Somatic Symptoms” factor revealed notable discrepancies, with the “Numbness or tingling” item displaying a correlation of 0.32 for the Brazilian sample, 0.53 for the Spanish sample, and 0.58 for the Portuguese sample.

Additionally, in the “Subjective Affective and Panic Symptoms” factor, the “Unable to relax” item obtained a lower correlation in the Brazilian sample (0.55) compared with the Spanish and Portuguese samples (0.67), and the “Fear of dying” item obtained a correlation of 0.48 in the Brazilian sample and 0.32 in the Spanish and Portuguese samples. Regarding this last result for the Spanish and Portuguese samples, no specific discussion was found for this item in the consulted literature. However, sociocultural factors related to the understanding of the expression “fear of dying” may be associated with that. Added to this, given that Brazil has higher rates of anxiety in its population compared with other countries, more pronounced symptoms of anxiety may manifest more acutely in this population, which may account for these discrepancies in factor loadings [3].

Divergent results were achieved with the CFA of the BAI, in which the unifactorial and two-factor solutions did not present good fit indices. The procedure that was adopted to sort the most appropriate model was the same as the aforementioned procedure. Again, the *χ*^2^ test result was significant (*p* < 0.001). Consistent with the findings of a recent meta-analysis with different psychometric properties of the BAI [29]. These authors found no evidence to support the factorial validity of the model that was proposed by Beck and Steer (1993) [30], with CFI values that ranged from 0.69 to 0.90 and RMSEA values that ranged from 0.04 to 0.14. However, the authors included only studies that analyzed English language versions of the BAI.

An MGCFA was conducted to assess the invariance of the two-factor solutions of the BDI-II and BAI for Brazilian, Spanish, and Portuguese undergraduate students. We present results from the lavaan package (version 0.6.14). A parsimonious examination of the characteristics of the models, together with a statistical summary, may facilitate an understanding of the invariant behavior of the BDI-II across configural, metric, and scalar levels. This interpretation is primarily supported by the optimal distribution of residuals as measured by the RMSEA in the configural model, which yielded an interval of 0.031 (0.027–0.035) and CFI value of 0.898, which are at the limit of acceptability. Additionally, the TLI value was marginally lower than what is currently recommended at 0.886. Furthermore, the CFI and TLI values increased significantly in the metric model (CFI = 0.954, TLI = 0.952), with a decrease in the RMSEA index = 0.20 (0.015–0.025). The behavior of these indices remained consistent, approaching 0.950 in the scalar model (CFI = 0.942, TLI = 0.943, RMSEA = 0.022 [0.018–0.026]).

The present study yielded divergent results for the BAI, in which it did not exhibit invariant behavior across any of the models at the scaled evaluation level. Specifically, at the configural level, the CFI and TLI values were 0.833 and 0.814, respectively, although the RMSEA value was within an acceptable range (0.041 [0.037–0.044]). These findings suggest instability in the factorial structure and other psychometric properties of the BAI among the countries that were included in the sample for the selected model.

With regard to the limitations of the present study, the research was performed among undergraduate students in three countries, thereby biasing the sampling. Additionally, due to the cross-sectional design that was adopted in the original study [22], it is not possible to understand the temporal changes of the phenomenon studied, which would be better verified through longitudinal investigations [39]. Another potential limitation was the lack of measuring bi-factor models of the BDI-II and invariance properties to gender, as proposed in a recent study [8]. One partial limitation of our analyses was the way we dealt with items of the measures. Because of convergence issues, we treated all items as continuous indicators. However, we used the DWLS estimator, which the current literature also recommends within the categorical analysis framework. Finally, we did not run an EFA to check which latent structure best fits the data collected, which will be carried out in future studies. Altogether, these limitations should be considered when attempting to generalize our findings.

With the COVID-19 pandemic, an increase in the prevalence of depression and anxiety has been detected [2]. Different degrees of anxiety and depression disorders are associated with extensive impairments in cognition that compromise daily living activities [40]. Tools are needed with robust psychometric properties. Our findings demonstrated that the BDI-II is suitable for clinical use, and its scoring can be considered invariant even between participants in different countries, such as Brazil, Spain, and Portugal.

To our knowledge, this is the first study that evaluated the invariance of the BDI-II and BAI among undergraduate students in Brazil, Portugal, and Spain. As discussed previously, because of the scarcity of studies on the psychometric properties of instruments in developing countries (e.g., Brazil), our findings will contribute to further development in this area. Our study also provided further evidence of the factorial structure of these popular and important instruments. Finally, to advance future research in the field, we strongly recommend investigating the invariance of these instruments with different factorial structures (e.g., bi-factor models) and genders.

## 5. Conclusions

The present study assessed the invariance of the BDI-II and BAI among undergraduate students from three different countries. The analyses revealed good psychometric properties of the BDI-II, whose invariance was fully supported by our analysis, whereas the BAI did not show similar results. The present results add to the evidence of the factor structure of these instruments.

## Figures and Tables

**Table 1:** Descriptive statistics of the BDI-II and BAI.

Item (BAI)	Mean	SD	Skewness	Kurtosis	Item (BDI-II)	Mean	SD	Skewness	Kurtosis
1. Numbness or tingling	0.41	0.63	1.39	1.17	1. Sadness	0.25	0.53	2.36	6.02
2. Feeling hot	0.69	0.75	0.74	−0.29	2. Pessimism	0.54	0.69	1.26	1.67
3. Wobbliness in legs	0.34	0.63	1.76	2.30	3. Feeling of failure	0.30	0.57	2.02	4.15
4. Unable to relax	0.89	0.89	0.66	−0.51	4. Feeling of guilt	0.37	0.59	1.50	2.08
5. Fear of worst happening	0.69	0.90	1.06	0.00	5. Feeling of guilt	0.49	0.61	1.13	1.65
6. Dizzy or lightheaded	0.28	0.61	2.23	4.29	6. Feeling of punishment	0.16	0.51	3.87	16.61
7. Heart pounding/racing	0.34	0.68	1.99	3.29	7. Disconformity with oneself	0.39	0.70	1.93	3.33
8. Unsteady	0.36	0.65	1.86	3.04	8. Self-criticism	0.79	0.72	0.71	0.37
9. Terrified or afraid	0.20	0.53	2.88	8.19	9. Suicidal thoughts	0.07	0.32	5.68	40.13
10. Nervous	1.04	0.88	0.50	−0.49	10. Crying	0.34	0.69	2.05	3.52
11. Feeling of choking	0.22	0.59	2.85	7.81	11. Agitation	0.54	0.69	1.32	1.99
12. Hands trembling	0.30	0.63	2.19	4.36	12. Loss of interest	0.43	0.62	1.46	2.37
13. Shaky/unsteady	0.17	0.47	3.14	10.56	13. Indecision	0.46	0.79	1.84	2.86
14. Fear of losing control	0.24	0.59	2.65	6.84	14. Devaluation	0.31	0.67	2.07	3.16
15. Difficulty breathing	0.22	0.57	2.88	8.30	15. Loss of energy	0.56	0.69	1.03	0.64
16. Fear of dying	0.18	0.56	3.42	11.57	16. Changes in sleeping habits	0.85	0.78	0.76	0.37
17. Scared	0.39	0.69	1.76	2.51	17. Irritability	0.43	0.64	1.51	2.33
18. Indigestion	0.55	0.77	1.27	0.85	18. Changes in appetite	0.53	0.71	1.31	1.52
19. Faint/lightheaded	0.14	0.44	3.41	12.29	19. Difficulties in concentration	0.76	0.80	0.65	−0.64
20. Face flushed	0.50	0.71	1.30	1.06	20. Tiredness or fatigue	0.53	0.69	1.23	1.30
21. Hot/cold sweats	0.37	0.66	1.77	2.62	21. Loss of sexual interest	0.14	0.45	3.93	17.19

SD, standard deviation.

**Table 2:** Descriptive results of demographic variables.

	Participants		Sex			Age			
Country	*n*	Total %	Male (%)	Female (%)	*p*	Total (Years [SD])	Male (Years [SD])	Female (Years [SD])	*p*
Brazil	315	16.1	149 (47)	166 (53)	0.37	22.8 (7.2)	22.1 (4.3)	23.4 (8.8)	0.11
Portugal	426	21.8	203 (48)	223 (52)	0.36	20.4 (1.6)	20.5 (1.6)	20.4 (1.7)	0.39
Spain	1210	62.1	384 (32)	825 (68)	<0.001	21.5 (3.0)	21.4 (3.5)	21.5 (2.8)	0.67
Total	1957	100.0	736 (38)	1214 (62)		21.5 (3.8)	21.3 (3.3)	21.5 (4.1)	

*n*, number of participants; %, absolute frequency.

**Table 3:** Standardized regression weights (factor loadings) of BDI-II items.

BDI-II Item	Factor Loadings			
	Brazilian	Spanish	Portuguese	Total
Factor 1: Somatic-Affective				
4. Loss of pleasure	0.52	0.62	0.63	0.72
10. Crying	0.56	0.48	0.42	0.60
11. Agitation	0.48	0.52	0.47	0.57
12. Loss of interest	0.56	0.58	0.63	0.69
13. Indecision	0.50	0.61	0.62	0.68
15. Loss of energy	0.64	0.67	0.65	0.76
16. Changes in sleeping habits	0.53	0.48	0.48	0.57
17. Irritability	0.49	0.60	0.60	0.67
18. Changes in appetite	0.52	0.52	0.49	0.59
19. Difficulties in concentration	0.51	0.59	0.60	0.64
20. Tiredness or fatigue	0.71	0.60	0.63	0.73
21. Loss of sexual interest	0.33	0.50	0.46	0.67
Factor 2: Cognitive				
1. Sadness	0.65	0.64	0.69	0.79
2. Pessimism	0.61	0.51	0.53	0.60
3. Feeling of failure	0.61	0.58	0.63	0.72
5. Feeling of guilt	0.54	0.57	0.53	0.63
6. Feeling of punishment	0.36	0.50	0.47	0.66
7. Disconformity with oneself	0.60	0.69	0.72	0.79
8. Self-criticism	0.58	0.57	0.59	0.63
9. Suicidal thoughts	0.55	0.36	0.34	0.68
14. Devaluation	0.64	0.66	0.65	0.82

**Table 4:** Confirmatory factor analysis of BDI-II for the total sample and by country.

Factor Structure	Sample	*χ* ^2^	*df*	CFI	TLI	RMSEA (90% CI)
BDI-II Two-Factors (Beck et al., 1996) [24]	Brazilian	258.002 *	188	0.922	0.912	0.035 (0.024–0.045)
	Portuguese	257.632 *	188	0.887	0.873	0.030 (0.020–0.039)
	Spanish	471.875 *	188	0.850	0.832	0.036 (0.032–0.040)
	Total	682.023 *	188	0.855	0.838	0.037 (0.034–0.040)

*df*, degrees of freedom; CFI, Comparative Fit Index; TLI, Tucker–Lewis Index; RMSEA, Standard Root Mean Square Error of Approximation; CI, confidence interval. * *p* < 0.001.

**Table 5:** Standardized regression weights (factor loadings) of BAI items.

BAI Item	Factor Loadings			
	Brazilian	Spanish	Portuguese	Total
Factor 1: Somatic Symptoms				
1. Numbness or tingling	0.32	0.53	0.58	0.51
2. Feeling hot	0.46	0.45	0.42	0.45
3. Wobbliness in legs	0.54	0.54	0.53	0.54
6. Dizzy or lightheaded	0.65	0.50	0.62	0.55
7. Heart pounding/racing	0.73	0.63	0.63	0.64
8. Unsteady	0.54	0.64	0.68	0.63
12. Hands trembling	0.59	0.65	0.65	0.64
13. Shaky/unsteady	0.61	0.64	0.62	0.64
17. Scared	0.72	0.62	0.61	0.63
19. Faint/lightheaded	0.45	0.49	0.56	0.51
20. Face flushed	0.45	0.38	0.45	0.38
21. Hot/cold sweats	0.48	0.51	0.49	0.50
Factor 2: Subjective Affective and Panic Symptoms				
4. Unable to relax	0.55	0.67	0.67	0.65
5. Fear of worst happening	0.73	0.69	0.69	0.69
9. Terrified or afraid	0.62	0.58	0.58	0.59
10. Nervous	0.70	0.69	0.70	0.69
11. Feeling of choking	0.55	0.68	0.67	0.66
14. Fear of losing control	0.75	0.55	0.54	0.58
15. Difficulty breathing	0.45	0.58	0.53	0.55
16. Fear of dying	0.48	0.32	0.32	0.35
18. Indigestion	0.48	0.52	0.56	0.52

**Table 6:** Confirmatory factor analysis of BAI for the total sample and by country.

Factor Structure	Sample	*χ* ^2^	*df*	CFI	TLI	RMSEA (90% CI)
BAI Two-Factors (Beck et al., 1993) [30]	Brazilian	287.705 *	188	0.807	0.784	0.043 (0.033–0.052)
	Portuguese	290.533 *	188	0.862	0.846	0.036 (0.028–0.044)
	Spanish	653.464 *	188	0.804	0.781	0.046 (0.042–0.050)
	Total	971.658 *	188	0.792	0.767	0.047 (0.044–0.050)

*df*, degrees of freedom; CFI, Comparative Fit Index; TLI, Tucker–Lewis Index; RMSEA, Scaled Root Mean Square Error of Approximation; CI, confidence interval. * *p* < 0.001.

**Table 7:** Multi-group CFA of BDI-II and BAI for Brazilian, Spanish, and Portuguese undergraduate students.

Instrument/Model	*χ* ^2^	*df*	CFI	ΔCFI	TLI	RMSEA (90% CI)
BDI-II Two-Factors						
Configural	911.549 *	564	0.898	-	0.886	0.031 (0.027–0.035)
Metric	757.522 *	602	0.954	0.056	0.952	0.020 (0.015–0.025)
Scalar	835.641 *	640	0.942	−0.012	0.943	0.022 (0.018–0.026)
BAI Two-Factors						
Configural	1151.617 *	564	0.833	-	0.814	0.041 (0.037–0.044)
Metric	945.792 *	602	0.902	0.069	0.898	0.040 (0.035–0.044)
Scalar	1221.724 *	640	0.863	−0.039	0.865	0.035 (0.031–0.038)

*df*, degrees of freedom; CFI, Comparative Fit Index; TLI, Tucker–Lewis Index; RMSEA, Root Mean Square Error of Approximation; CI, confidence interval. * *p* < 0.001.

## Data Availability

All codes and data can be found at https://osf.io/pzskj/ accessed on 5 May 2023.
